# The MS Risk Allele of CD40 Is Associated with Reduced Cell-Membrane Bound Expression in Antigen Presenting Cells: Implications for Gene Function

**DOI:** 10.1371/journal.pone.0127080

**Published:** 2015-06-11

**Authors:** Judith Field, Fernando Shahijanian, Stephen Schibeci, Australia and New Zealand MS Genetics Consortium (ANZgene), Laura Johnson, Melissa Gresle, Louise Laverick, Grant Parnell, Graeme Stewart, Fiona McKay, Trevor Kilpatrick, Helmut Butzkueven, David Booth

**Affiliations:** 1 Multiple Sclerosis Division, Florey Institute of Neuroscience and Mental Health, University of Melbourne, Melbourne, Victoria., Australia; 2 Centre for Immunology and Allergy Research, Westmead Millennium Institute, University of Sydney, Sydney, New South Wales, Australia; 3 Melbourne Brain Centre at the Royal Melbourne Hospital, University of Melbourne, Parkville, Australia; 4 Department of Medicine, University of Melbourne, Melbourne, Victoria, Australia; 5 Melbourne Neuroscience Institute, University of Melbourne, Melbourne, Victoria, Australia; INSERM, FRANCE

## Abstract

Human genetic and animal studies have implicated the costimulatory molecule CD40 in the development of multiple sclerosis (MS). We investigated the cell specific gene and protein expression variation controlled by the CD40 genetic variant(s) associated with MS, i.e. the T-allele at rs1883832. Previously we had shown that the risk allele is expressed at a lower level in whole blood, especially in people with MS. Here, we have defined the immune cell subsets responsible for genotype and disease effects on CD40 expression at the mRNA and protein level. In cell subsets in which CD40 is most highly expressed, B lymphocytes and dendritic cells, the MS-associated risk variant is associated with reduced CD40 cell-surface protein expression. In monocytes and dendritic cells, the risk allele additionally reduces the ratio of expression of full-length versus truncated CD40 mRNA, the latter encoding secreted CD40. We additionally show that MS patients, regardless of genotype, express significantly lower levels of CD40 cell-surface protein compared to unaffected controls in B lymphocytes. Thus, both genotype-dependent and independent down-regulation of cell-surface CD40 is a feature of MS. Lower expression of a co-stimulator of T cell activation, CD40, is therefore associated with increased MS risk despite the same CD40 variant being associated with reduced risk of other inflammatory autoimmune diseases. Our results highlight the complexity and likely individuality of autoimmune pathogenesis, and could be consistent with antiviral and/or immunoregulatory functions of CD40 playing an important role in protection from MS.

## Introduction

The CD40 gene has been previously identified as a risk gene for multiple sclerosis (MS) [1–4] and other autoimmune diseases, including Graves’ disease (GD) [5–8], rheumatoid arthritis (RA) [9–12], systemic lupus erythematosus (SLE) [13] and Crohn’s disease (CD) [3]. CD40 is an important co-stimulatory molecule expressed on the surface of a variety of antigen presenting cells (APCs) including dendritic cells (DCs) and B-lymphocytes, as well as cells of the innate immune system such as macrophages and microglia. CD40 has previously been shown to play a role in the development of animal models of autoimmune demyelinating disease. Depletion by antagonistic antibodies [14–16] or ablation (gene knock-out) [17] of CD40 expression results in amelioration of disease, highlighting the importance of the secondary activation signal in these inflammatory models. More recently, over-expression of CD40 in the thyroid has been shown to lead to spontaneous induction of hyperthyroidism in a murine model [18].

While GD and RA are associated with the major allele at rs1883832 (C) associated with increased CD40 expression [5,6] and therefore might be predicted to enhance a pro-inflammatory environment/response [19], the risk allele for MS at rs1883832 (T, minor allele) is associated with reduced CD40 expression [1,20]. Although there are many SNPs in linkage disequilibrium (LD) with rs1883832, it is possible that rs1883832 itself mediates the functional effects of this LD block. It is located at -1bp of the transcription start site (TSS) within the Kozak consensus sequence, in which the major C allele has been shown to lead to enhanced efficiency of translation of the corresponding gene transcript [5,6]. However it is entirely possible that other SNPs in the LD block may be contributing to or causing the functional effect driving association with disease susceptibility. In addition, the effects of individual SNPs on expression of CD40 may, as for other immune cell genes, be highly dependent on context (i.e. inflammation) and cell subset.

Previous studies have suggested that CD40 expression is increased at the mRNA level in peripheral blood mononuclear cells (PBMC) in MS compared to healthy/non-MS controls [21], but is not different in cultured B lymphocytes or monocytes at the protein level [22]. However, these studies involved small cohorts of varying disease duration and disease course (including secondary progressive MS and primary progressive MS), and/or subjects concurrently treated with disease-modifying therapies, all of which could potentially effect CD40 expression.

In this study, we used a relatively large cohort of untreated MS patients and unaffected controls to investigate the effect of genotype on expression of peripheral blood mononuclear cell types that produce the highest levels of CD40: B lymphocytes and monocytes. As other antigen presenting cells (APCs) are rare in blood, but the APCs from secondary lymphoid organs and tissues have the highest expression of CD40 of all subsets analysed in published databases (www.immgen.org, www.biogps.org), we also used *in vitro* differentiation of monocytes to produce dendritic cells representative of these cell types. Further, we examined the effect of disease on CD40 expression in B-lymphocytes and monocytes freshly isolated from the peripheral blood of MS patients with relapsing-remitting MS (RRMS) compared to age- and sex-matched healthy controls. Our findings implicate lowered cell-surface CD40 levels in the development of MS, and should lead to further mechanistic investigations with potential therapeutic implications.

## Materials and Methods

### Subject recruitment and demographics

Whole blood samples were collected between 8am and 1pm and processed within 3 hours for the isolation of peripheral blood mononuclear cells (PBMCs; EDTA tubes); or stored at -20 degrees for whole-blood RNA (PAXgene tubes).

MS patients were recruited according to the following criteria:—definite relapsing-remitting MS (RRMS) according to McDonald criteria or Clinically Isolated Syndrome (CIS), aged between and inclusive of 18–65 years, not currently on immunomodulatory therapy for MS, or none within in the last three months and no other concurrent autoimmune disease. In our cohort, MS patients were between the ages of 21 and 54 years (38.4± 7.7). The mean number of years from diagnosis at the time of sample collection was 6.0 ± 7.0. Neurological outcome was assessed using the Kurtzke Expanded Disability Status Scale (EDSS). The median EDSS was 2.0 (mean EDSS 1.9 ± 1.6). There were 18 females and 3 males within our MS cohort. Unaffected controls were aged between and inclusive of 18–65 years of age and had no personal history of neurological disease or autoimmune disease. Two independent healthy control cohorts were used. Cohort 1 was composed of 56 females and 28 males, aged 37.46 (± 10.1) and cohort 2: 32 females and 17 males (entirety or subsection of cohort 2 used in figures as described). All MS patients and healthy controls recruited for this study were Caucasian.

### Ethics Statement

Ethical approval for this project was obtained from the Eastern Health Research Ethics Committee (Melbourne) and the Western Sydney Local Health District Human Ethics Committee. All participants provided written consent.

### Cell subset purification

Immune cell subsets were purified from PBMCs isolated from MS patients and controls by either Ficoll (GE Healthcare) or Histopaque (Invitrogen) density gradient separation. CD4, CD8, CD4+CD45RO+ and CD4+CD45RA+ T cells, T regulatory cells (Tregs) (CD4+CD25hi), B lymphocytes (CD19+) NK cells (CD3-CD56+), monocytes (CD14+), plasmacytoid dendritic cells (CD303+CD304+CD123+CD11c-) and myeloid dendritic cells (CD19+CD1c-hi) were purified using magnetic bead separation as previously described [23]. For monocyte subsets, a monocyte enrichment kit (StemCells) was used prior to CD14 purification as per the manufacturers’ instructions (Human CD14 Microbeads, Miltenyi Biotec, Germany). Th1, Th2 and Th17 subsets were differentiated *in vitro* from CD4+CD45RA+ as previously described [24]. DC1 and DC2s were generated *in vitro* from monocytes by sequential incubation with IL-4 and GMCSF, LPS and either IFNγ (DC1) or IFNβ (DC2) as previously described [23]. Purified subsets were stored in RLT buffer (Qiagen) or Cells-to-signal Buffer (Ambion) for subsequent RNA extraction. Purity of the cellular subsets was determined by flow cytometry.

### Flow cytometric analysis

Fresh PBMC from MS patients and healthy controls were analysed by flow cytometry using flow cytometry antibodies purchased from Miltenyi Biotec (Germany), including CD14-PE (TUK4) and CD16-FITC (VEP13) for monocytes and CD19-FITC (LT19), IgG-PE (IS11-3B2.2.3), IgD-PE (IgD26), CD27-PECy5 (M-T271), CD38-PE (IB6), and CD24-biotin (32D12/anti-biotin-PerCP for B lymphocyte subsets. Regulatory B cells were identified as CD19+CD38hiCD24hi using CD19-FITC, CD38-PE and CD24-biotin/antibiotin-PerCp antibodies as described by the Mauri laboratory [25,26]. CD40 expression was determined in PBMCs or purified/cultured subsets by comparison of CD40-APC (HB14) or CD40-PE (HB14) to an isotype control (Mouse IgG_1_ clone IS5-21F5). Labelled cells were analysed using a CyAn ADP analyzer (Beckman Coulter) and the data analysed using WEASEL v3.0.

### Genotyping and gene expression of CD40

DNA was extracted from whole blood as previously described [1]. DNA was genotyped for rs1883832 using the Taqman SNP genotyping assay C_11655119 (Applied Biosystems), or by restriction fragment length polymorphism: PCR amplification using oligonucleotides 5’- ACAGCAAGATGCGTCCCTAAAC- 3’ and 5’- CTTCCCTTTCCTTCTCATTCCC- 3’ followed by enzyme digestion with *Nco1* (Promega), generating products of 114 bp and 226 bp for the C allele and 340 bp for the T allele at rs1883832.

RNA was extracted from purified cells using the RNeasy Mini Kit (Qiagen) including DNAse treatment. RNA from whole blood was collected using PAXgene blood RNA tubes (PreAnalytiX, Switzerland) and total mRNA extracted using the PAXgene Blood RNA Kit (Qiagen, Germany). Total expression of CD40 was determined by SYBR green qRT-PCR using the following primers- forward: 5’-GCAGGGGAGTCAGCAGA-3’; reverse: 5’-TTCCTTCCCTTT CCTTCTCA-3’; and the housekeeping gene GAPDH as previously described [20], or by next generation mRNA sequencing (RNA-Seq) analysis as previously described [27]. The percentage of CD40 transcript encoding the full-length protein (FL) was determined by PCR amplification of a cDNA region spanning CD40 exon 4 to exon 10 using primers:—forward 5’-CAGACACCATCTGCACCTGT-3’ and reverse, 5’-AATTGATCTCCTGGGGT TCC-3’. Molarity of the largest splice form (~400bp, encoding the full-length isoform) as a proportion of all isoforms expressed was determined by electrophoresis and UV detection (Bioanalyzer, Agilent Technologies) as previously described [24]. The expression level of CD40 isoforms was determined for a series of immune cell subsets using RNAseq (annotated using Ensembl build GRCh37.62) as previously described [23].

### Statistical Analysis

Statistical analysis was performed using GraphPad Prism 5. Comparisons between groups were made by unpaired t-test or Mann-Whitney U test as appropriate. Box and whisker plots depict maximum and minimum values, median and interquartile range.

## Results

### In peripheral blood immune cells, B-lymphocytes express the highest level of CD40 mRNA and protein

**Fig 1:**
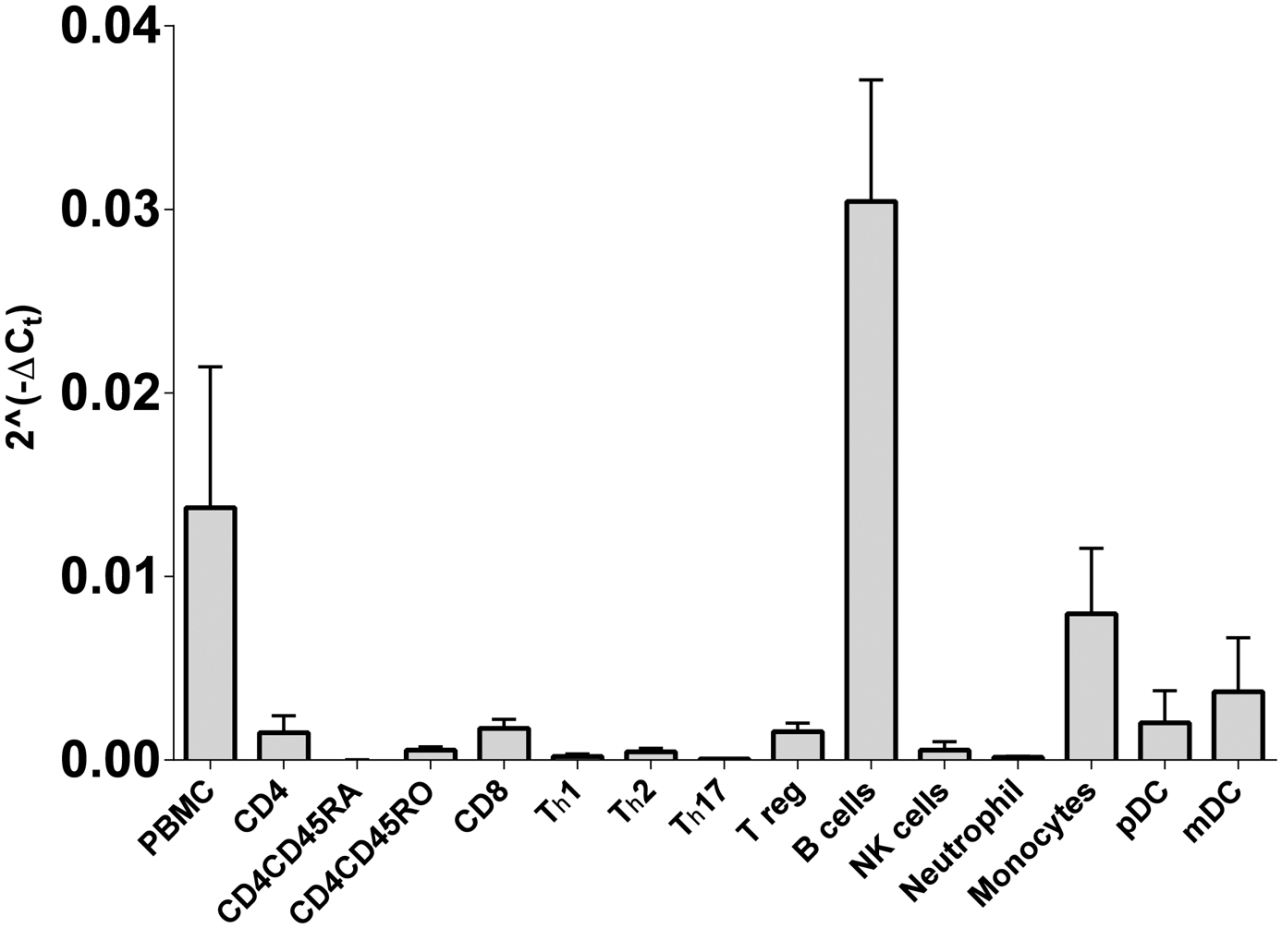
CD40 mRNA expression in peripheral blood immune cell subsets. CD40 mRNA expression was determined by RT-PCR in freshly purified immune cell subsets or *in vitro* differentiated subsets (Th1, Th2, Th17; differentiated from fresh CD4CD45RA) from healthy controls (n = 3, or n = 2 for pDC).

In our earlier work [20] we demonstrated that CD40 mRNA expression was genotype dependent in whole blood. Here we compared expression in cell subsets purified from blood to confirm the likely source of these differences in mRNA expression. As expected, of the common cell subsets found in blood, B-lymphocytes have the highest level of CD40 mRNA, with monocytes and dendritic cells also contributing (Fig 1). In an RNAseq analysis of immune cell subsets we previously conducted [28], several mRNA isoforms were identified in key subsets of immune cells, including transcript encoding the full length protein (dominant) and those lacking exon 5 and/or exon 6 that encode for the transmembrane region, resulting in the translation of soluble CD40 protein.

### Expression of the CD40 MS risk allele correlates with reduced CD40 levels on B-cells

**Fig 2:**
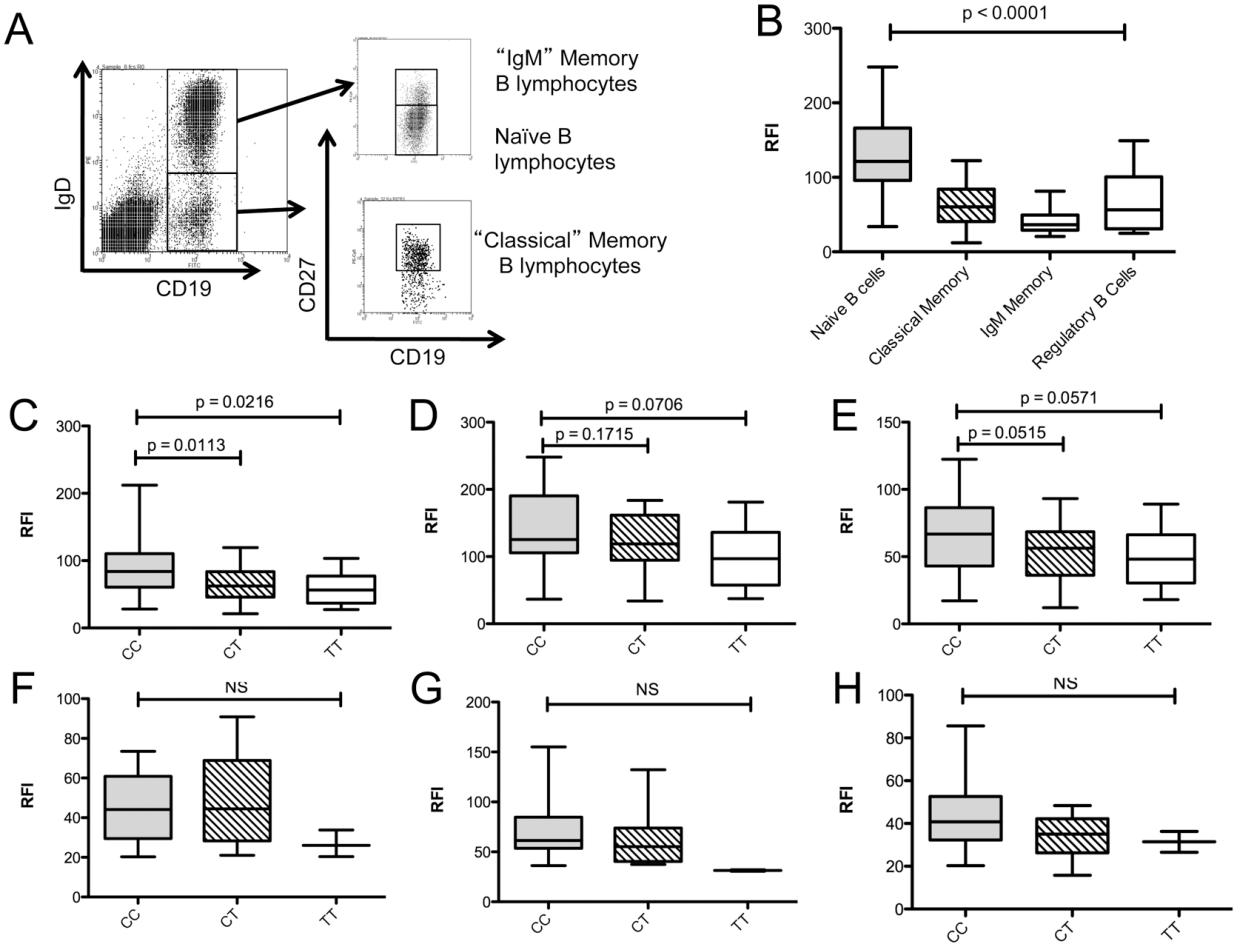
Genotype dependent CD40 protein expression in peripheral B-lymphocyte subsets of MS patients and healthy controls. B lymphocyte subsets from healthy controls (n = 86) and MS patients (n = 21) were identified by flow cytometry (A) and CD40 expression determined relative to an isotype control (relative fluorescence intensity; RFI). Regulatory B cells were identified as CD19+CD38hiCD24hi (data not shown) (B). Association of rs1883832 genotype with CD40 expression in healthy controls (CC = 49, CT = 27, TT = 10) was examined in total B lymphocytes (C), naïve B-lymphocytes (D) and classical memory B-lymphocytes (E), and in total B lymphocytes (F), naïve B lymphocytes (G) and classical B lymphocytes (H) of MS patients (CC = 12, CT = 7, TT = 2). p values were determined by Mann-Whitney U test comparison of each group.

B-lymphocytes from healthy controls and MS patients were analysed *ex vivo* for expression of surface CD40 protein using flow cytometry. B-lymphocytes were defined by forward and side scatter (FSC/SSC) and expression of CD19 on the cell surface, and B-lymphocyte sub-populations were defined by the presence or absence of the surface markers IgD and CD27. These were analysed for CD40 expression compared to an isotype control (Fig 2A). Naïve B cells expressed significantly more CD40 on the cell surface compared to classical memory, IgM memory B and regulatory B lymphocyte subsets (Fig 2B). Total B-lymphocytes showed a genotype-dependent reduction in the surface expression of CD40 (Fig 2C); with homozygous CC individuals (n = 49) expressing 30% more total CD40 on the cell surface compared to CT (n = 27; p = 0.0113) and TT (n = 10; p = 0.0216) individuals. The surface expression of CD40 on naïve B cells (CD19+IgD+CD27-) was not significantly associated with genotype (Fig 2D; CC vs. CT p = 0.1715, CC vs. TT p = 0.0706), while classical memory B cells (Fig 2E, CD19+IgD-CD27+) demonstrated a trend towards a genotype-dependent CD40 expression profile (CC vs. CT p = 0.0515; CC vs. TT p = 0.0571). No significant genotype-dependent expression effects were observed in total B cells (Fig 2F; CC vs. CT p = 0.2511; CC vs. TT p = 0.3924)), naïve B cells (Fig 2G; CC vs. CT p = 0.5701, CC vs. TT p = 0.1271)) or classical memory B cells (Fig 2H; CC vs. CT p = 0.2511, CC vs. TT p = 0.3924) isolated from MS patients. In this study, the genotype effect on CD40 mRNA expression measured in whole blood by RNA-Seq did not reach significance (data not shown).

### CD40 is under expressed on MS patient B-lymphocytes independent of the CD40 risk allele effect

**Fig 3:**
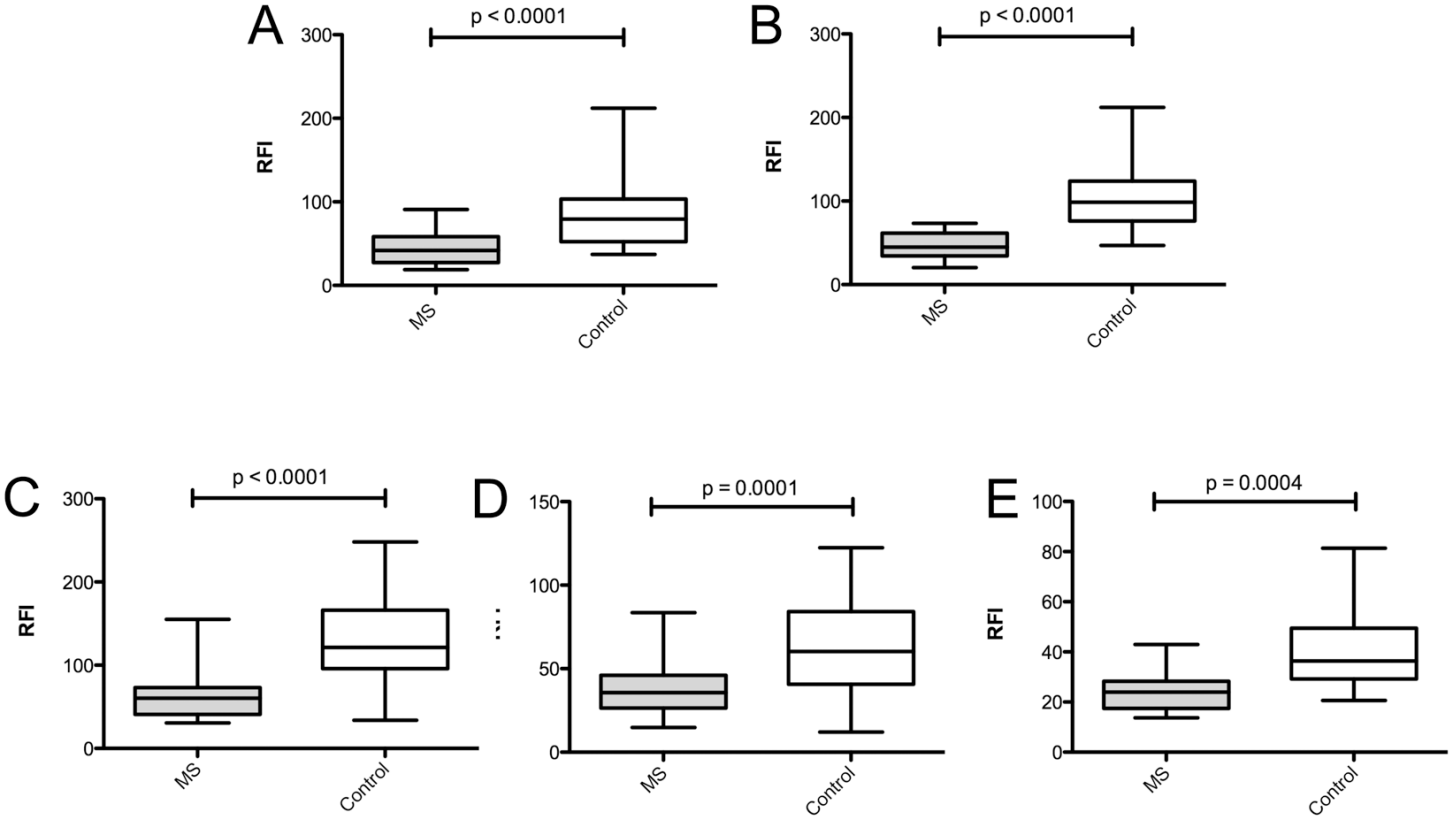
CD40 protein is under- expressed in B lymphocytes of MS patients. B lymphocyte subsets from healthy controls (n = 86) and MS patients (n = 24) were identified by flow cytometry and CD40 expression determined relative to an isotype control (relative fluorescence intensity; RFI). Surface levels of CD40 were compared in total B-lymphocytes (A), B lymphocytes from rs1883832 CC individuals (B; n = 49 healthy controls, n = 12 MS patients), naïve B lymphocytes (C), classical memory B lymphocytes (D) and IgM memory B lymphocytes (E). P-values were determined using Mann—Whitney test.

Comparison of B lymphocyte expression of CD40 in MS patients failed to show a significant effect of genotype on surface CD40 expression levels in total B lymphocytes (Fig 2F), naïve B lymphocytes (Fig 2G) or classical memory B lymphocytes (Fig 2H). However, comparison of cell surface CD40 expression on B-lymphocytes between healthy controls and MS patients (Fig 3) showed that CD40 expression was significantly lower in MS patients compared to healthy controls in all CD19+ B-lymphocytes (Fig 3A; p <0.0001), as well as in the naïve B lymphocytes\ (Fig 3C; p <0.0001), classical memory B-lymphocyte (Fig 3D; p = 0.0001) and IgM memory B lymphocyte subsets (Fig 3E; p = 0.0004). A subset comparison of patients and unaffected controls homozygous for the rs1883832 C allele (CC) also demonstrated a significant decrease in CD40 expression on the total B—lymphocytes of MS patients compared to controls (Fig 3B; p <0.0001) The relative proportions of total B-lymphocytes and subsets as a percentage of total white cells were not affected by genotype or phenotype (data not shown).

### CD40 is expressed at significantly lower levels in “classical” monocytes compared to “intermediate” and “non-classical” monocytes

**Fig 4:**
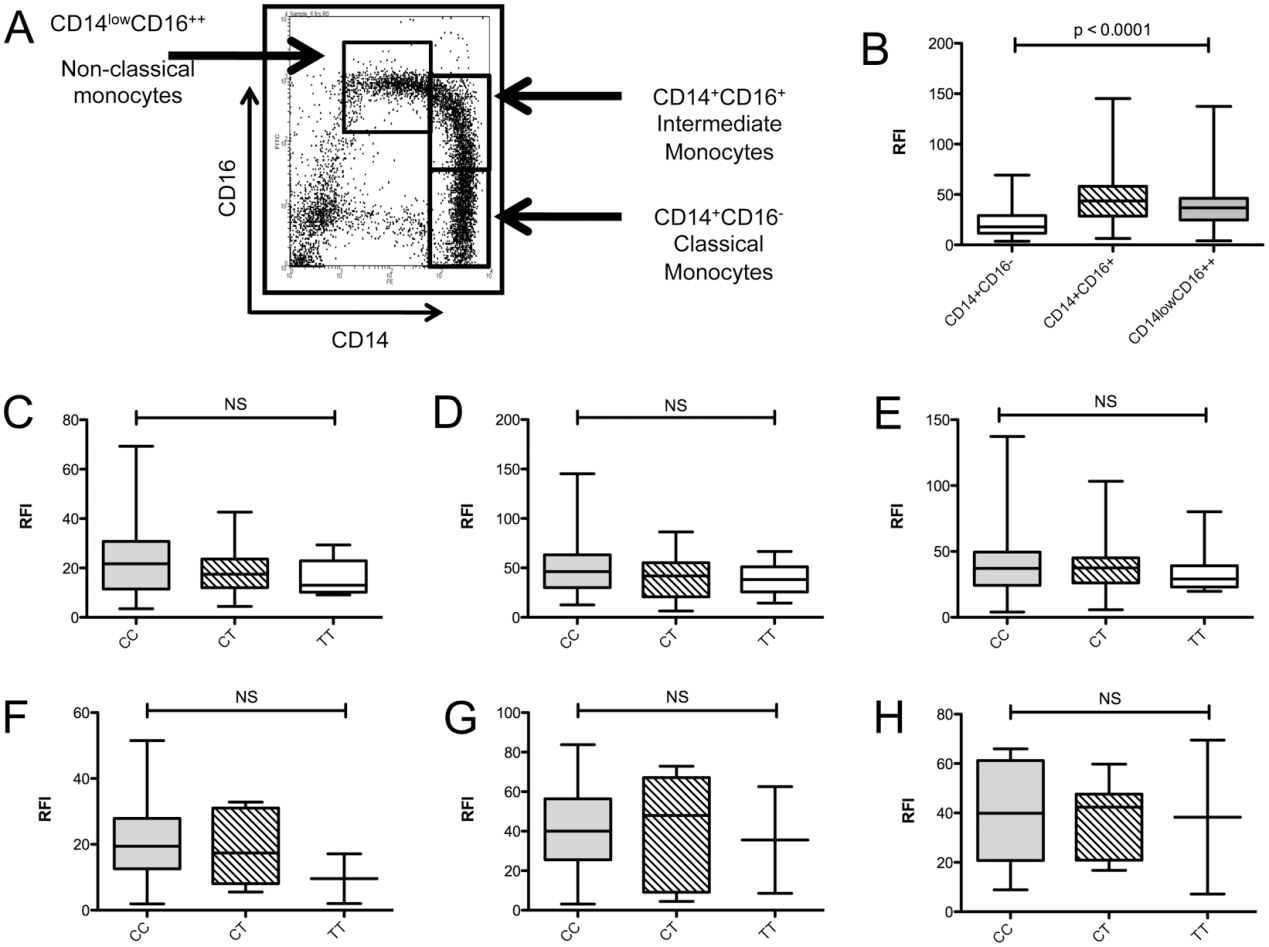
Genotype dependent CD40 protein expression in peripheral monocyte subsets of MS patients and healthy controls. Monocyte subsets were identified by flow cytometry (A) and CD40 expression determined relative to an isotype control (relative fluorescence intensity; RFI) (B). Association of rs1883832 genotype with CD40 expression was examined in classical CD14+CD16- (C), intermediate CD14+CD16+ (D) and non-classical CD14lowCD16++ monocytes (E) from healthy controls (CC = 49, CT = 27, TT = 10), and classical CD14+CD16- (F), intermediate CD14+CD16+ (G) and non-classical CD14lowCD16++ monocytes (H) from MS patients (CC = 12, CT = 7, TT = 2). P—values were determined by Mann-Whitney test.

Monocytes from MS patients and healthy controls defined by FSC/SSC profiles and CD14 positivity were analysed for expression of CD40. Classical monocytes were defined as CD14+CD16-, intermediate monocytes as CD14+CD16+, and non-classical monocytes as CD14low, CD16++ (Fig 4A). The proportion of monocytes and the individual monocyte subtypes were not affected by risk-genotype or phenotype (data not shown). CD14+ CD16- classical monocytes expressed significantly lower levels of CD40 on the cell surface compared to the intermediate and non-classical monocyte subtypes in both healthy controls and MS (Fig 4B).

### CD40 expression is not significantly affected by genotype or phenotype in monocyte subsets

There was no significant difference in the level of CD40 expression in monocytes between MS and controls (Fig 4C). In addition, there were no genotype-dependent effects on CD40 expression in healthy controls (Fig 4D).

### The CD40 MS risk-allele is under expressed in dendritic cell subsets

**Fig 5:**
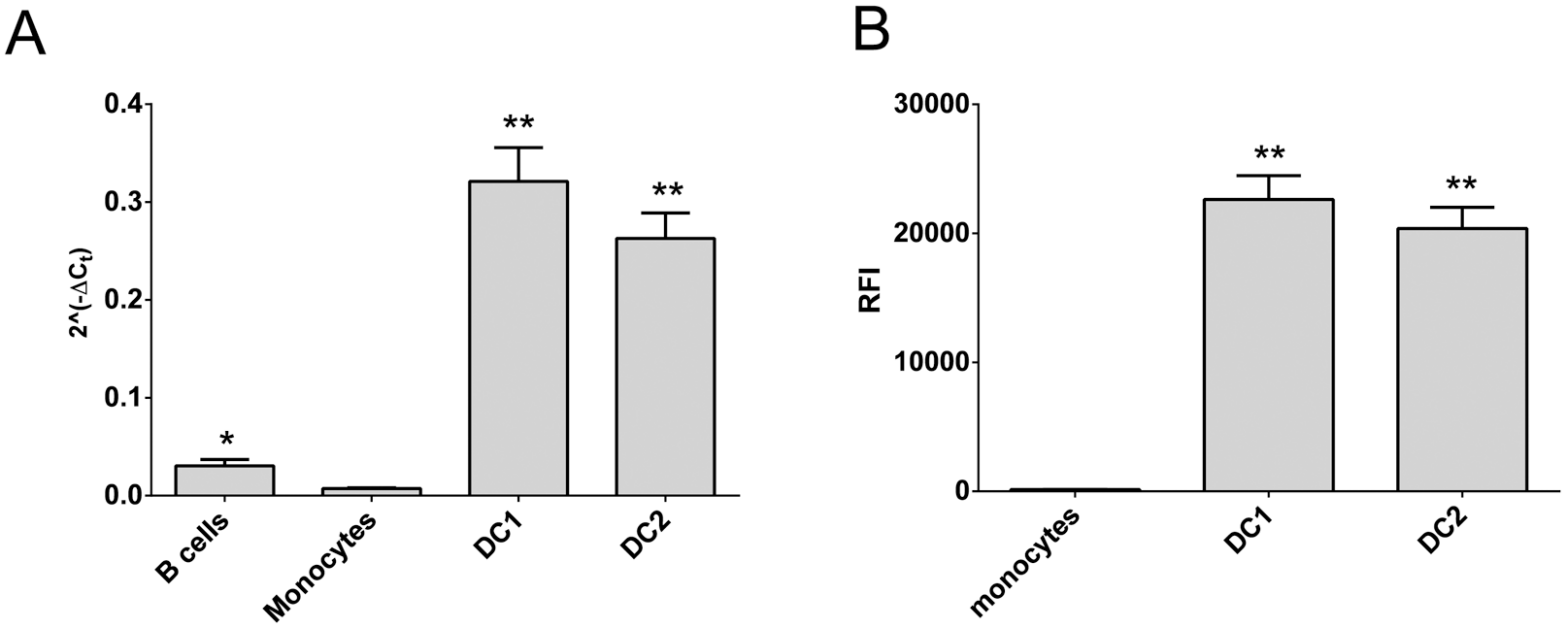
CD40 expression is higher in differentiated dendritic cell subsets. CD40 expression was determined in freshly purified immune cell subsets (B cells, monocytes) or *in vitro* differentiated dendritic cells (DC1, DC2) from healthy controls. Gene expression by RT-PCR (A; n = 49) and relative fluorescence intensity (RFI) by flow cytometry (B; n = 41) are shown; *significantly different from monocytes and DCs; **significantly different from B cells and monocytes (A) or from monocytes (B); p < 0.05 by Mann-Whitney test.

**Fig 6:**
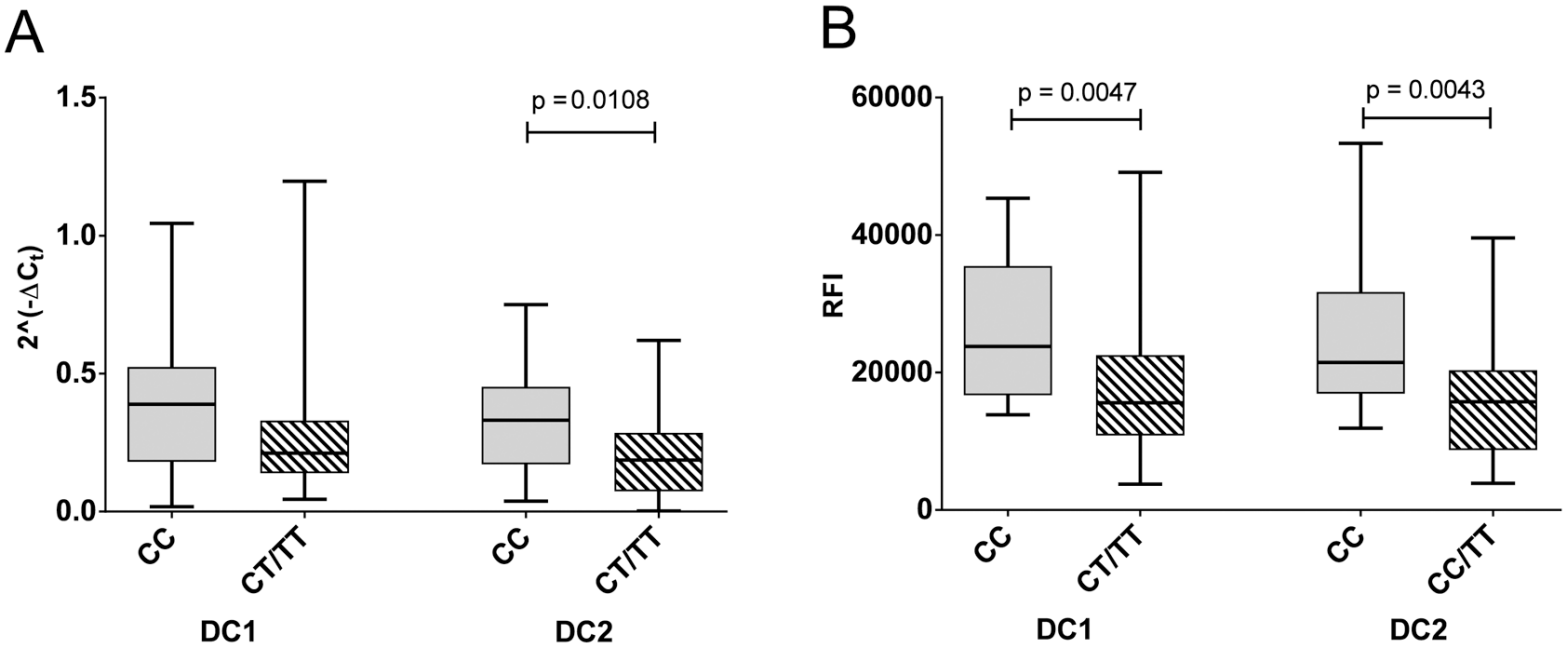
The CD40 risk allele is under expressed in dendritic cell subsets. Association of rs1883832 genotype with CD40 expression was determined in *in vitro* differentiated dendritic cells (DC1, DC2) from healthy controls (non-carriers, CC, or carriers, CT/TT, of the risk allele). Gene expression by RT-PCR (A; n = 49) and relative fluorescence intensity (RFI) by flow cytometry (B; n = 41) are shown; p values by Mann-Whitney test.

Dendritic cells are the major antigen presenting cells. However, DCs from whole blood are not typical of those in the secondary lymphoid organs and tissues, which are thought to drive T cell activation in autoimmune diseases [29]. Fortunately DCs representative of tissue DCs can be differentiated from monocytes, and have been verified as inflammatory (DC1) or tolerogenic (DC2) on the basis morphology and IL12p40 and IL10 mRNA and protein expression [23]. These DCs express much higher levels of CD40 mRNA and protein than monocytes and B cells (Fig 5). In these cells, CD40 expression was genotype dependent, with reduced expression of the risk allele at the mRNA level in DC2s (Fig 6A, p < 0.011) and at the protein level in both DC phenotypes (Fig 6B; p < 0.0047, DC1s; p < 0.0043, DC2s).

### Lower proportions of the full-length isoform from the CD40 MS risk-allele

**Fig 7:**
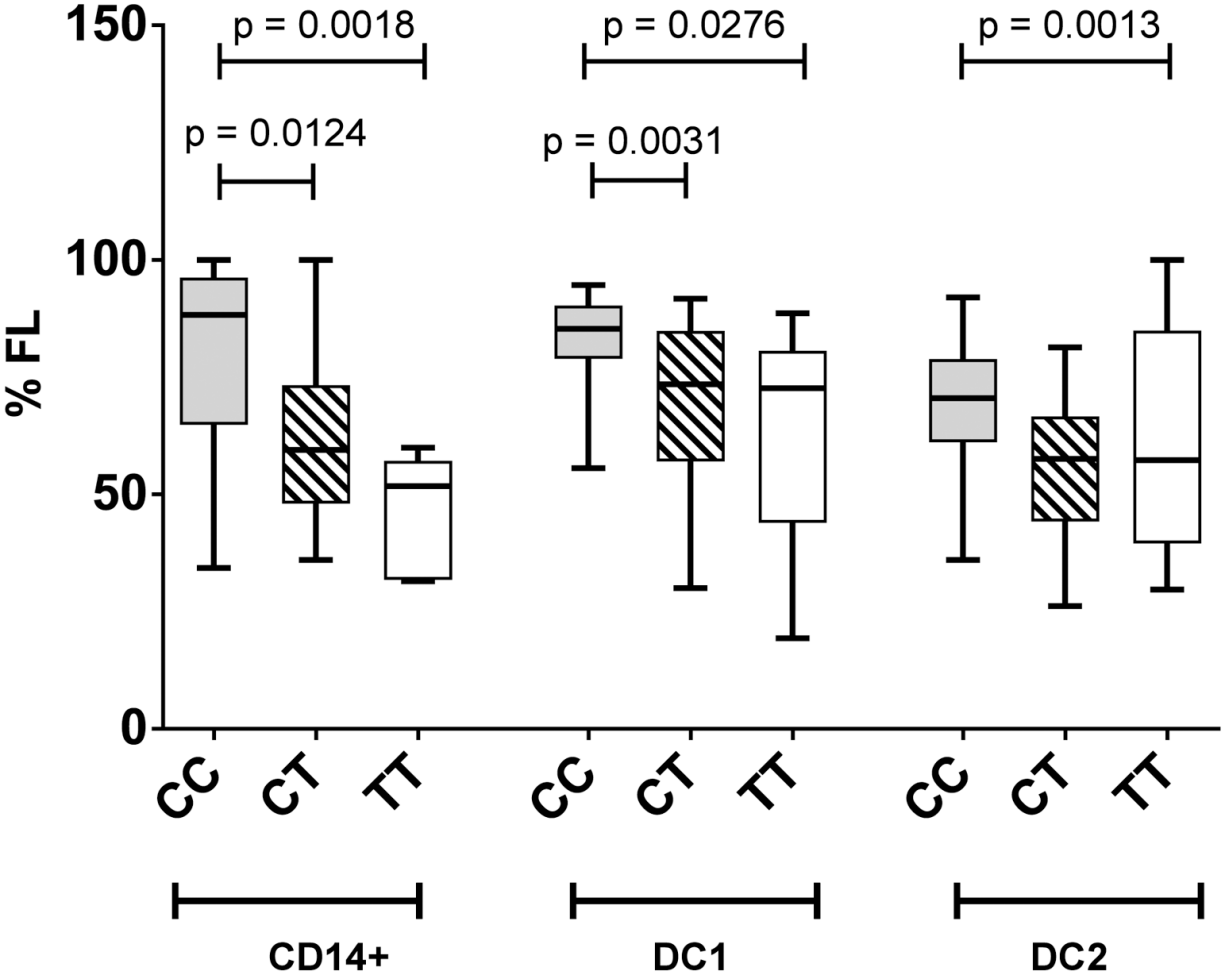
Lower proportions of the full-length isoform expressed from the CD40 risk allele in monocytes and dendritic cells. Association of rs1883832 genotype (CC, TC, TT) with the proportion of full-length isoform of CD40 (%FL) expressed in *in vitro* differentiated dendritic cells (DC1, DC2) from healthy controls (n = 49). Molar ratios of isoforms were quantitated by RT-PCR and amplification of a region spanning CD40 exon 4–10, followed by electrophoretic separation and fluorescent detection (Bioanalyzer, Agilent); p values by Mann-Whitney test.

**Fig 8:**
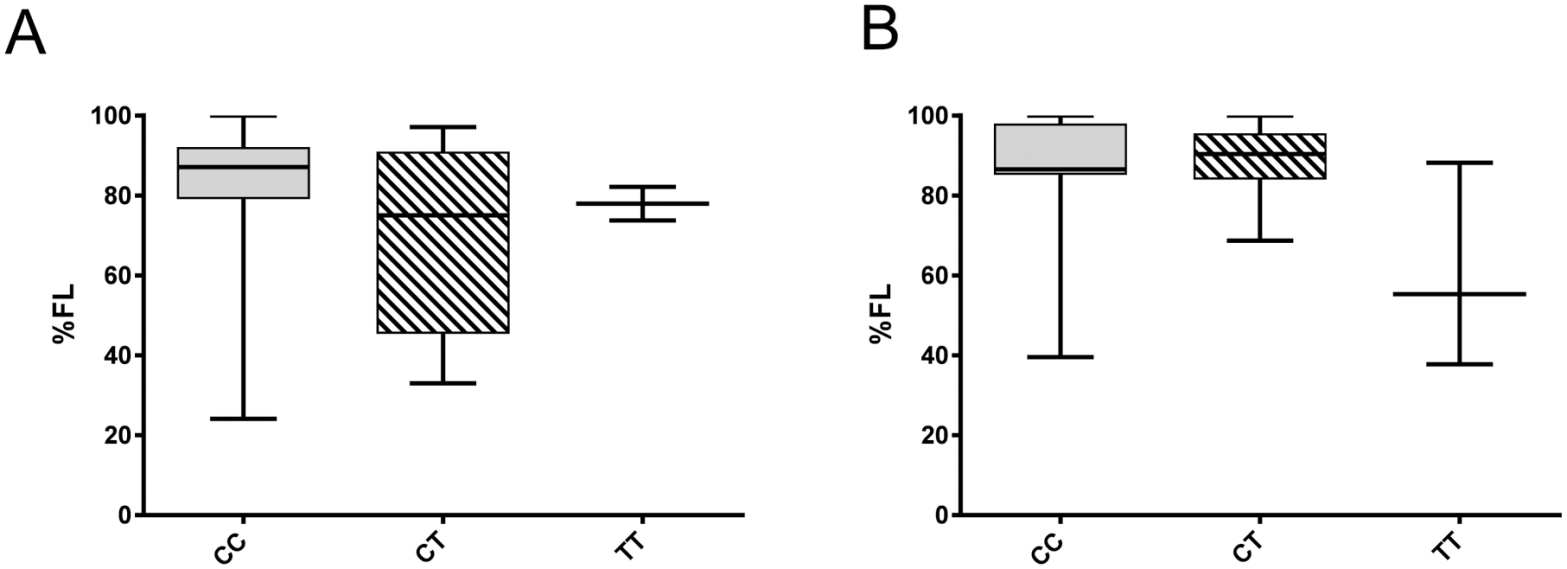
Proportions of the full-length isoform expressed from the CD40 risk allele in whole blood. Association of rs1883832 genotype (CC, TC, TT) with the proportion of full-length isoform of CD40 (%FL) expressed in whole blood from healthy controls (A; n = 38) and MS (B; n = 32). Molar ratios of isoforms were quantitated by RT-PCR and amplification of a region spanning CD40 exon 4–10, followed by electrophoretic separation and fluorescent detection (Bioanalyzer, Agilent). Trends were observed for CC > CT in controls (p < 0.13) and for CT > TT in MS, (p < 0.056); p values by Mann-Whitney test.

Greater splicing of the CD40 risk allele was evident with a lower percentage of the full length mRNA isoform expressed in DCs and monocytes compared to expression levels in DC carrying at least one protective allele (Fig 7; p < 0.0020, monocytes; p < 0.0014, DC1s; p < 0.0026, DC2s). A similar trend was evident in whole blood in healthy controls (CC > CT; p < 0.13) and MS (CT > TT; p < 0.056; Fig 8). As CD40 isoform usage was affected by the MS risk genotype, we sought common SNPs located between exon 4 and exon 8 that might affect splicing. All SNPs identified as inherited in strong LD with rs6074022 in the 1000 genome project and located between exon 4 and exon 8 (rs73115010, rs66815221, rs73622651, rs6074028, rs3746821, rs11569333) were intronic and in regions unlikely to affect splicing, as assessed with the Human Splicing Finder tool [30]. The minor allele frequency of the exonic SNPs from exon 4 to exon 8 was less than 4%, so unlikely to be driving the genotype association. This included 3 SNPS (rs369901991, rs371997367, rs144600981) calculated in Ensembl to potentially affect splicing.

## Discussion

In this study we show an MS risk genotype-dependent reduction of CD40 cell-surface protein in B-lymphocytes and polarised dendritic cells. This is paralleled by lower levels of CD40 mRNA production from the risk genotype in these cells, and an increased relative proportion of isoforms encoding the secreted form of CD40. In addition, and for the first time, we show that the level of CD40 protein expression is significantly reduced in B-lymphocytes isolated from MS patients compared to healthy controls, independent of risk genotype. This is consistent with our previous findings that whole blood CD40 mRNA was reduced in carriers of the risk genotype, and that the effect of genotype on expression was enhanced in MS [20]. These results also point towards additional factors leading to the down-regulation of CD40 protein expression in MS patients besides CD40 genotype, and thus implicate lower cell surface CD40 protein expression in the complex pathogenesis of MS.

However, our findings are in contrast to previous studies that have shown no difference in either the mRNA or protein levels of CD40 expression between MS patients and controls [21,22]. These previous cohorts were of varying disease phenotype, including patients in the progressive phase of disease, varying relapse status and with a wider range of disease duration [21,22] possibly capturing, at least for those with relapse, a more inflammatory state with concomitantly higher CD40 expression, thus masking the reduced CD40 expression we observed in our cohort of patients with stable relapsing-remitting disease.

The opposite genetic association of CD40 rs1883832 with increased susceptibility to MS, but with protection from RA and GD are intriguing and point to distinct roles for CD40 in the pathogenic processes in these autoimmune diseases. Our studies here and previously [20], as well as studies in RA and GD [5,6] have consistently demonstrated an association of the T allele with reduced CD40 expression. The association of protection from RA and GD with lower expression of a T cell activation gene fits with a primarily costimulatory role of CD40 supporting the autoimmune inflammatory process, and supported by animal models in which CD40 inhibition reduces inflammation. In contrast, the association of protection from MS with higher expression of CD40 may suggest that these inflammatory processes are protective in MS, and/or that thymic tolerogenic processes mediated by CD40 are of greater importance in protection from MS than CD40-mediated autoimmune inflammatory processes are in disease initiation or propagation.

It is intriguing that Epstein Bar virus (EBV), long implicated in MS pathogenesis [31], encodes a homologue of human CD40, This protein is expressed in infected B cells, and constitutively signals, promoting B cell proliferation [32]. Relative susceptibility to EBV may be dependent on competition between human and EBV CD40/CD40L signaling. Notably though, a genome wide association study of SNPs with EBNA-1 antibody levels did not implicate CD40 [33]. This would suggest any CD40 genotype associations with EBV susceptibility, or EBV contribution to MS, may be independent of EBNA-1 antibody levels.

Tolerogenic responses mediated by CD40 stimulation include naïve B lymphocyte- mediated stimulation of T cells leading to the expansion of regulatory T cells [34]. In addition, naïve B lymphocytes (and regulatory B cells found within the naïve B lymphocyte pool) have been shown to produce regulatory cytokines such as IL-10 upon stimulation with CD40L [35]. In addition to these peripheral immunoregulatory mechanisms, cortical and medullary thymic epithelial cells (cTECS and mTECs) express functional CD40 [36], which has been shown to be essential for the establishment of the mTEC microenvironment leading to tolerance to self-antigens [37]. The role of Vitamin D/UVR driven immunomodulation [38,39], mediated by APCs, may also provide an additional link between reduced CD40 expression and increased MS risk, with under-expression of CD40 by DCs leading to the failure of the protective effects of increased Vitamin D/UVR.

A decrease in CD40 expression by these cell types could plausibly lead to a failure of tolerance/immunomodulatory mechanisms mediated by CD40 stimulation, and by extension, a failure of protection from the development of MS in subsequent years.

While CD40 expression is known to be increased in thyroid tissue in GD [40], no examination has been made of either the genotype-dependent effects of CD40 expression in GD patients, or CD40 expression by peripheral immune cells in the context of disease. In SLE, a risk-genotype dependent correlation in CD40 expression level in B lymphocytes has been identified, present in both patients and healthy controls [13], however there is no apparent genotype-independent effect in CD40 expression levels between SLE patients and healthy controls. Certainly, the genotype-independent decrease in CD40 expression by peripheral immune cells in MS is a unique and novel finding, and leads to many questions as to the reasons for this decrease, as well as the subsequent effects. Is this decrease a result of “exhaustion” of CD40 expression in MS similar to that of CD8 “exhaustion” in chronic viral infection [41], or is it a result of homeostatic down-regulation in the context of inflammation?

The absence of a known genetic variant in the exon 4 to 8 region that would drive the observed risk genotype dependent splicing differences could indicate that either a novel variant exists which has yet to be included in the 1000genome and ensemble databases, or that mRNA splicing is regulated from outside this region, as has been described for other genes [42]. Although increased production of soluble isoforms could be expected to reduce signaling through reducing both cell surface expression and offering alternative ligand binding, soluble receptors have also been shown to potentially increase signaling through increasing the half-life of ligands (e.g. potentially for soluble CD40L) [43], and further work is required to ascertain if soluble CD40 levels are indeed relatively increased in MS patients, as these data suggest. The net effect of the risk haplotype on expression may actually a be gain of function.

Further work is needed to understand the consequences of decreased CD40 expression on B lymphocyte and dendritic cells in antiviral/immunoregulatory functions in demyelinating disease, and what drives reduced expression of CD40 in B lymphocytes in established MS. Defining the genotype-independent but disease-dependent changes in B lymphocyte and dendritic cell CD40 levels identified in this study could uncover protective roles for CD40 in MS that fundamentally distinguish its pathogenesis from that of other autoimmune diseases.
